# Features of tumor texture influence surgery and outcome in intracranial meningioma

**DOI:** 10.1093/noajnl/vdaa113

**Published:** 2020-09-10

**Authors:** Thomas Sauvigny, Franz L Ricklefs, Lena Hoffmann, Raphael Schwarz, Manfred Westphal, Nils Ole Schmidt

**Affiliations:** 1 Department of Neurosurgery, University Medical Center Hamburg-Eppendorf, Hamburg, Germany; 2 Department of Neurosurgery, University Medical Center Regensburg, Regensburg, Germany

**Keywords:** meningioma, microsurgery, Simpson grading, surgical outcome, tumor texture, vascularization

## Abstract

**Background:**

Texture-related factors such as consistency, vascularity, and adherence vary considerably in meningioma and are thought to be linked with surgical resectability and morbidity. However, data analyzing the true impact of meningioma texture on the surgical management is sparse.

**Methods:**

Patients with intracranial meningioma treated between 08/2014 and 04/2018 at our institution were prospectively collected for demographics, clinical presentation, histology, and surgical treatment with related morbidity and extend of resection. Tumor characteristics were reported by the surgeon using a standardized questionnaire including items such as tumor consistency, homogeneity, vascularization, and adherence to surrounding neurovascular structure and analyzed for their impact surgical outcome parameters using univariate and logistic regression analyses.

**Results:**

Tumor texture-related parameters of 300 patients (72.3% female) with meningioma were analyzed. Meningioma localizations were grouped into 3 different cohorts namely convexity, skull base, and posterior. Postoperative occurrence of a neurological deficit (transient 23.0%; permanent 6.1%) was associated with the duration of surgery (*P* = .001), size of tumor (*P* = .046), tumor vascularization (*P* = .015), and adherence to neurovascular structures (*P* = .002). Coherently, the duration of surgery (mean 230.99 ± 101.33 min) was associated with size of tumor (*P* < .0001), vascularization (*P* < .0001), and adherence (*P* < .0001). Similar associations were recapitulated in subgroup analyses of different tumor localizations. Noteworthy, tumor rigidity had no significant impact on time of surgery and neurological outcome.

**Conclusions:**

Our analysis demonstrates that tumor texture has an impact on the surgical management of meningioma and provides data that tumor vascularization and adherence are significant factors influencing surgical outcome whereas the influence of tumor consistency has less impact than previously thought.

**Key Points**

- Meningioma tumor texture parameters are associated with the occurrence of neurological deficits.
- Tumor consistency has less impact on time of surgery and neurological outcome than previously thought.

Standard therapy for meningioma is the surgical resection with the potential of cure if the tumor can be completely resected including the dural origin.^1^ However, in many cases this is not feasible and while radiation therapy is of limited value, there are currently no meaningful chemotherapeutic options.^2^

The extend of resection (EOR) as reflected in the Simpson Grading Scale is the strongest predictor for tumor recurrence and a major determinant for prognosis.^3,4^ Given the primary and central significance of surgery for the further disease course in meningioma patients the prediction of factors influencing surgical resection and the natural history are of utmost importance. Although never studied in detail it is widely accepted that the texture of meningioma is a major determinant for EOR and the risk for surgical morbidity.^5–8^ Several studies have attempted to evaluate MR imaging for the prediction of tumor texture^8–11^ but with conflicting results. Features of tumor texture include consistency (also referred to as hardness, rigidity, or stiffness), adherence, or infiltration of neurovascular structures and tumor vascularization.^7^ Meningiomas display a highly variable inter- and intratumoral heterogeneity in this regard. Meningioma with a hard consistency and high adherence to blood vessels, cranial nerves, and/or to the surrounding brain parenchyma represents a significant challenge for surgical removal. In these cases, the attempt for complete resection is paralleled by a significant increase of morbidity and often results in an incomplete resection which is especially relevant in anatomical locations with a high density of essential neurovascular structures such as the skull base.^6^ Therefore, the prediction of texture-related parameters before surgery is necessary in order to develop a risk adapted therapeutic strategy which has essential implications for patient counseling and to allocate surgical logistics if not a “wait & see” strategy is decided.

Importance of the StudyTexture-related factors such as consistency, vascularity and adherence vary considerably in meningioma and are thought to be linked with surgical resectibility and morbidity. Here we analyse 300 patients with meningioma for their tumor-texture related parameters by using a standardized questionnaire. Postoperativ occurrence of a neurological deficit was associated with the duration of surgery, size of tumor, tumor vascularization and adherence to neurovascular structures, whereas tumor consistency had no significant impact. Our analysis demonstrates that tumor texture has a significant impact on the surgical management of meningioma highlighting the need for preoperative prediction of tumor texture to optimize surgical strategy and risk assessment.

However, although the relationship between texture and surgical morbidity is well accepted in the neurosurgical community a relationship based on scientific analysis has not been established so far. In this study, we aim to objectify the role of meningioma texture on surgical morbidity and clinical outcome parameters. We aim to provide data of tumor texture-related parameters which are necessary for the further development of predictive tools such as MR imaging.

## Methods

### Study Population

Three hundred patients admitted from August 2014 to April 2018 with histopathological confirmed meningioma and complete hospital records were enrolled in this prospective study. Five patients with extremely rare meningioma localizations (eg, intraventricular) were excluded from further analysis. Patients demographics, clinical presentation, imaging data, histology, and surgical treatment with related morbidity and EOR were recorded. In line with the EANO guidelines for the diagnosis and treatment of meningiomas,^1^ all patients received a postoperative MRI scan 3 months after the procedure and were seen in our outpatient clinic. Subsequently, regular follow-up visits with annual MRI controls were conducted. In cases of WHO II and III meningioma routine MRI scans and clinical follow-up visits were performed at least in 6-month intervals.

### Data Analysis

Tumor texture characteristics were reported by the surgeon using a standardized questionnaire adapted from Zada et al.^12^ and expanded to include items such as tumor rigidity, homogeneity, vascularization, and adherence to surrounding neurovascular structure. All surgeons were informed about the questionnaire prior to the study to ensure a uniform handling directly after surgery (within 24 h). The levels of textural features were defined by a semiquantitative grading system that was based on the surgeons’ assessment of intraoperative aspects and necessary surgical maneuvers and tools for tumor removal seen in Supplementary Material (Questionnaire).

**Figure 1.**
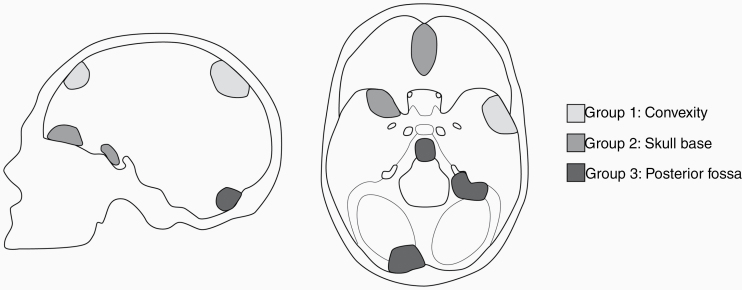
Illustration of Meningioma subgroups.

The entire cohort was analyzed with regard to morbidity and EOR. For a more detailed presentation and in order to allow an analysis based on a generalized anatomical context, meningiomas were also grouped into the following localizations: convexity (group 1: including falcine meningiomas, lateral sphenoid wing meningiomas), skull base (group 2: including meningiomas of the sellar and parasellar region, medial sphenoid wing meningiomas), and posterior fossa (group 3: including meningiomas of the cerebello-pontine angle, tentorial meningiomas) (Figure 1).

Neurological deficits were assessed during the hospital stay, at discharge and during follow-up at our outpatient department. Transient morbidity was defined as a neurological deficit that resolved itself during the hospital stay or at follow-up visits. Morbidity was classified as permanent when not resolved after a follow-up time of 3 months. The preoperative and postoperative neurological status was assessed using the modified Rankin Scale (mRS).

Simpson grading^3^ was assessed during surgery and reported by the surgeon. Tumor size was determined by the largest diameter in preoperative MRI in axial, coronary, or sagittal alignment. The duration of the surgery was recorded by a semiautomatic medical documentation system.

Additionally, we evaluated the preoperative MRIs in 119 patients and analyzed for correlations between standard MR imaging and textural features. Since the data derived from various MR scanners of external referral centers, we calculated ratios of signal intensities of the meningioma and healthy brain tissue (gray matter) on the contralateral hemisphere in standard MR imaging sequences (T2-weighted intensity meningioma/gray matter, T1-weighted intensity meningioma/gray matter, contrast-enhanced T1-weighted intensity meningioma/gray matter and T2/T1 ratio of the meningioma).

Mean intensity values of the 2 regions of interest (ROIs) were calculated from the datasets in the respective MRI sequence. ROIs were defined as tumor tissue and distant gray matter. Using Spearman’s rank correlation these parameters were tested for an association with the WHO grade, a neurological deterioration on the mRS, tumor vascularization, adherence, and consistency as indicated in the questionnaire.

Informed consent was obtained from all participants. The study was reported to the local ethic committee (PV4904) and was performed in accordance with the ethical standards laid down in the Declaration of Helsinki and local authority regulations.

### Statistical Analysis

Statistical analyses of the data were performed by a univariate analysis using Kruskal–Wallis test or ANOVA tests depending on the scale of the measurements and homogeneity of variances, to examine correlations between the parameters using IBM SPSS Statistics 22 (IBM Corporation, Armonk, NY). Nominal data were compared using Fisher’s exact test or the Chi2-test. To assess the relationship between the MR imaging findings and clinical and tumor texture characteristics a Spearman’s rank correlation was calculated.

A multivariate regression analysis, including parameters showing a *P* value <0.1 in univariate analysis, was calculated for the entire cohort to assess parameters associated with morbidity and EOR. The level of statistical significance was set at *P* < .05.

## Results

### Demographics and Clinical Presentation

A total of 300 patients fulfilled the inclusion criteria described above and were included in this analysis. 73.9% (*n* = 218) of the patients were female. Mean age of the cohort was 56.63 ± 13.16 years (range 24–86 years). Meningiomas were grouped into 3 different cohorts depending on their localization, namely convexity (44.1%), skull base (38.3%), and posterior fossa (17.6%) (Figure 1 and Table 1). Histological WHO grading of meningiomas varied between all location groups with more high-grade meningiomas in the convexity group (Table 1). The EOR as reflected by the Simpson grade was higher for meningiomas of the convexity and posterior fossa group (*P* = .004) (Table 1). Surgery of skull base meningiomas resulted in a higher rate of permanent deficits (10.8%; a.e. paresis, ataxia, speech deficit, cranial nerve palsy) (Table 1). The mortality rate in the full cohort was 1%. 10.16% of all patients were included in our study due to tumor recurrence or progression of a previously treated meningioma.

**Table 1.** Study Cohort Characteristics

Characteristic	Overall	Convexity	Skull base	Posterior fossa	*P*
	*n* = 295	*n* = 130 (44.1%)	*n* = 113 (38.3%)	*n* = 52 (17.6%)	
Gender	218	89	88	34	.773
(Female)	73.9%	69.0%	79.3%	66.7%	
Age	58.63 ± 13.165	58.46 ± 14.177	58.54 ± 12.249	59.91 ± 12.538	.0581
	Range 24–86	Range 24–84	Range 30–86	Range 34–82	
Max. diameter (cm)	3.27 ± 1.58	3.63 ± 1.63	2.87 ± 1.52	3.36± 1.51	**.045**
	Range 0.46–9.2	Range 0.5–9.20	Range 0.46–8.2	Range 0.70–7.60	
WHO I	251 (85.1%)	99 (76.2%)	102 (90.3%)	50 (96.2%)	**.003**
WHO II	43 (14.6%)	30 (23.1%)	11 (9.7%)	2 (3.8%)	
WHO III	1 (0.3%)	1 (0.8%)	0 (0.0%)	0 (0.0%)	
Simpson grade	3	2	3	2	**.004**
	Range 1–5	Range 1–5	Range 1–5	Range 1–5	
Deficit	85 (28.8%)	38 (29.5%)	32 (28.8%)	15 (29.4%)	**.016**
Transient	68 (23.5%)	35 (27.3%)	20 (18.0%)	13 (25.5%)	
Permanent	17 (5.7%)	3 (2.3%)	12 (10.8%)	2 (3.9%)	

Bold numbers highlight significant values.

### Postoperative Morbidity

Univariate analysis of surgical- and tumor texture-related parameters in all patients revealed that the presence of a postoperative neurological deficit was associated with a longer duration of surgery (*P* = <.0001), higher tumor vascularization (*P* = .016), and adherence of the tumor to the surrounding tissue (*P* = <.0001) (Table 2). No association was found for age, tumor size, and EOR and consistency of the tumor. As meningiomas in different locations are often linked with specific surgical challenges we grouped the meningiomas in 3 different locations. Similarly, surgical time (*P* = <.0001) and tumor vascularization (*P* = .003) as well as adherence (*P* = .001) (Table 2) were significantly associated with a postoperative neurological deficit in the convexity group. These trends were also to some extend visible for the groups of skull base and posterior fossa meningiomas; however, this did not reach statistical significance. The multivariate analysis including relevant parameters from the univariate analysis demonstrated that for overall cohort of meningiomas the surgical time (odds ratio [OR] = 1.003, CI = 1.00–1.01, *P* = .013) and tumor adherence (OR = 1.443, CI = 1.00–1.02, *P* = .014) were predictive for a postoperative neurological deficit. Similar results were found for meningiomas of the convexity group but not in the skull base or posterior fossa group (Table 2). Based on the proven relevance of adherence, we additionally examined whether there was a correlation between adherence and histological parameters. None of routine histopathological parameters (WHO grade, histopathological subgroup according to the WHO classification 2017 or proliferation index) correlated with adherence.

**Table 2.** Postoperative Morbidity

Parameter		All locations			Convexity			Skull base			Posterior fossa		
		No deficit (*n* = 206)	Deficit (*n* = 85)	*P*	No deficit (*n* = 91)	Deficit (*n* = 38)	*P*	No deficit (*n* = 79)	Deficit (*n* = 32)	*P*	No deficit (*n* = 36)	Deficit (*n* = 15)	*P*
Surgery	Gender female (*n* = 291)	150	61	.886	60	29	.299	64	24	.606	26	8	.221
		(72.8%)	(71.8%)		(65.9%)	(76.3%)		(81.0%)	(75.0%)		(72.2%)	(53.3%)	
	Age (years)	59.02 ± 13.23	58.12 ± 13.03	.791	58.72 ± 14.26	57.92 ± 14.153	.771	58.86 ± 12.66	57.75 ± 11.32	.666	60.10 ± 11.96	59.44 ± 14.266	.791
		Range (24–86)	Range (26–83)		Range (24–84)	Range (26–83)		Range (30–86)	Range (41–79)		Range (36–82)	Range (34–79)	
	Duration of surgery (min)	218.06 ± 91.55	263.31 ± 116.928	**<.0001**	175.70 ± 54.67	236.37 ± 113.014	**<.0001**	247.28 ± 105.13	272.34 ± 120.99	.279	261.03 ± 91.04	312.27 ± 105.547	.087
		Range (60–870)	Range (108–630)		Range (60–360)	Range (108–545)		Range (90–870)	Range (130–630)		Range (130–480)	Range (164–571)	
	Max. diameter (cm)	3.17 ± 1.56	3.59 ± 1.63	.05	3.52 ± 1.60	3.88 ± 1.67	.271	2.73 ± 1.45	3.22 ± 1.65	.146	3.17 ± 1.56	3.59 ± 1.63	.380
		Range (0.46–8.6)	Range (1.10–9.20)		Range (0.50–8.6)	Range (1.70–9.20)		Range (0.46–8.2)	Range (1.10–7.50		Range (0.46–8.6)	Range (1.10–9.20)	
	Preoperative mRS	1.25 ± 0.92	1.28 ± 0.983	.774	1.08 ± 0.79	1.26 ± 1.13	.288	1.35 ± 0.975	1.25 ± 0.803	.593	1.44 ± 1.03	1.40 ± 0986	.887
		Range (0–4)	range (0–5)		Range (0–3)	range (0–5)		Range (0–4)	range (0–4)		Range (0–4)	range (0–4)	
	Simpson grade	2.09 ± 0.991	2.27 ± 0.923	.039	1.67 ± 0.82	1.78 ± 0.71	.462	2.47 ± 1.05	2.59 ± 0.8	.462	2.33 ± 0.862	2.80 ± 1.09	.118
		Range (0–5)	Range (0–5)		Range (1–5)	Range (1–4)		Range (1–5)	Range (1–4)		Range (1–5)	Range (2–5)	
	Recurrent surgery	21	8	1	8	2	.722	10	3	.544	3	3	.343
		(10.2%)	(9.4%)		(8.8%)	(5.3%)		(12.7%)	(9.4%)		(5.9%)	(5.9%)	
Tumor texture	Vascularization	1.60 ± 0.65	1.82 ± 0.73	**.016**	1.35 ± 0.57	1.76 ± 0.79	**.003**	1.80 ± 0.63	1.88 ± 0.61	.534	1.81 ± 0.71	1.87 ± 0.83	.850
	Heterogeneity	22.5%	23.5%	.878	24.7%	28.9%	.661	20.3%	21.9%	1	22.2%	13.3%	.703
	Adherence	1.81 ± 0.90	2.21 ± 0.91	**<.0001**	1.62 ± 0.77	2.21 ± 0.94	**.001**	2.01 ± 0.90	2.16 ± 0.77	.382	1.86 ± 1.01	2.33 ± 1.18	.157
	Consistency	2.74 ± 1.07	2.80 ± 1.08	.651	2.71 ± 1.03	3.06 ± 1.07	.100	2.67 ± 1.12	2.59 ± 0.80	.182	2.97 ± 1.08	2.60 ± 1.50	.247

Bold numbers highlight significant values.

### Surgical Resectability

The EOR as reflected by the Simpson grading is a major predictive factor for tumor recurrence in meningiomas.^13^ We therefore divided all patients into those with a Simpson grade 1–3 resection and those that were partially resected (Simpson grade 4–5). Univariate analysis of all patients revealed that the duration of surgery (*P* = .006), recurrent surgery (*P = <*.0001), tumor vascularization (*P* = .016), heterogeneity (*P* = .005), and adherence (*P* < .0001) were associated with an incomplete meningioma resection (Table 3). Univariate analysis of the subgroups based on location showed that within the convexity group tumor vascularization (*P* = .003) and tumor heterogeneity (*P* = .039), while in the skull base group tumor heterogeneity (*P* = .007) and adherence (*P* = < .0001) and in the posterior fossa group operation time (*P* = .003) and tumor adherence (*P* = .030) were associated with an incomplete resection (Table 3). In a multivariate regression model including statistical relevant parameters from the univariate analysis demonstrated that for the whole cohort recurrent surgery (OR = 0.210, CI = 0.09–0.51, *P* = .001) and tumor adherence (OR = 0.420, CI = 0.029–0.61, *P* = <.001) were predictive for an incomplete resection. Analysis of the subgroups revealed that tumor texture-related parameters had a significant impact on EOR (Table 4). In the convexity group tumor vascularization (OR = 0.269, CI = 0.09–0.81, *P* = .019) had a significant negative impact on EOR, while in the skull base group tumor heterogeneity (OR = 0.257, CI = 0.08–0.81, *P* = .021) and adherence (OR = 0.254, CI = 0.13–0.51, *P* = <.001) were significantly related to a reduced EOR. In the posterior fossa group surgical time (OR = 0.991, CI = 0.98–1.00, *P* = .029) and adherence (OR = 0.448, CI = 0.24–0.98, *P* = .044) to neurovascular structures were predictive for Simpson grade 4 and 5 (Table 4).

**Table 3.** Surgical Resectability

Parameter		All locations			Convexity			Skull base			Posterior fossa		
		Simpson ≤3 (*n* = 254)	Simpson >3 (*n* = 40)	*P*	Simpson ≤3 (*n* = 123)	Simpson >3 (*n* = 6)	*P*	Simpson ≤3 (*n* = 90)	Simpson >3 (*n* = 23)	*P*	Simpson ≤3 (*n* = 41)	Simpson >3 (*n* = 11)	*P*
Surgery	Gender female (*n* = 291)	183	31	.568	85	4	1	71	19	.780	27	8	1
		(72.0%)	(77.5%)		(69.1%)	(66.7%)		(78.9%)	(82.6%)		(65.9%)	(72.7%)	
	Age (years)	58.89 ± 13.44	57.94 ± 11.89	.671	58.71 ± 14.54	56.16 ± 7.06	.671	58.60 ± 12.41	58.44 ± 12.30	.956	60.10 ± 12.35	57.86 ± 13.85	.605
		Range (24–83)	Range (34–83)		Range (24–84)	Range (44–65)		Range (30–86)	Range (41–83)		Range (36–86)	Range (41–83)	
	OP duration (min)	225.85 ± 99.51	273.45 ± 106.32	**.006**	193.02 ± 82.92	213.33 ± 37.71	.553	256.20 ± 111.81	251.26 ± 100.982	.848	257.76 ± 86.1	352.64 ± 103.625	**.003**
		Range (60–870)	Range (130–630)		Range (60–545)	Range (150–247)		Range (90–870)	Range (130–630)		Range (130–480)	Range (233–571)	
	Max. diameter (cm)	3.32 ± 1.58	3.12 ± 1.61	.492	3.60 ± 1.60	4.26 ± 2.17	.376	2.99 ± 1.59	2.39 ± 0.99	.107	3.19 ± 1.37	3.94 ± 1.69	.138
		Range (0.46– 9.20)	Range (1.00–7.60)		Range (0.50– 9.20)	Range (1.60–7.10)		Range (0.46– 8.20)	Range (1.00–4.30)		Range (0.70– 6.00)	Range (2.00–7.60)	
	Preoperative mRS	1.24 ± 0.92	1.40 ± 1.01	.304	1.11 ± 0.90	1.67 ± 1.03	.146	1.30 ± 0.92	1.43 ± 0.95	.533	1.46 ± 0.98	1.18 ± 1.17	.419
		Range (0–5)	Range (0–4)		Range (0–5)	Range (0–3)		Range (0–4)	Range (0–4)		Range (0–4)	Range (0–4)	
	Deficit	73	11	1	36	1	.672	27	5	.451	10	5	.135
		(29.1%)	(28.2%)		(29.5%)	(16.7%)		(30.7%)	(21.7%)		(24.4%)	(50.0%)	
	Recurrent surgery	18	11	**<.0001**	9	1	.390	5	8	.001	4	2	.595
		(7.1%)	(27.5%)		(7.3%)	(16.7%)		(5.6%)	(34.8%)		(9.8%)	(18.2%)	
Tumor texture	Vascularization	1.63 ± 0.68	1.90 ± 0.67	**.016**	1.44 ± 0.66	2.17 ± 0.41	**.003**	1.80 ± 0.62	1.87 ± 0.63	.626	1.83 ± 0.70	1.82 ± 0.87	.885
	Heterogeneity	19.8%	40.0%	**.008**	24.0%	66.7%	**.039**	14.4%	43.5%	**.007**	19.5%	18.2%	1
	Adherence	1.80 ± 0.82	2.73 ± 1.11	**<.0001**	1.76 ± 0.86	2.33 ± 0.82	.086	1.86 ± 0.71	2.78 ± 1.00	**<.0001**	1.80 ± 0.93	2.82 ± 1.47	**.030**
	Consistency	2.73 ± 1.05	2.99 ± 1.17	.259	2.82 ± 1.06	2.57 ± 0.94	.546	2.52 ± 0.97	3.17 ± 1.07	**.015**	2.90 ± 1.16	2.82 ± 1.47	.764

Bold numbers highlight significant values.

**Table 4.** Regression Analysis

Variables	Overall			Convexity			Skull base			Posterior fossa		
	*P*	OR	CI	*P*	OR	CI	*P*	OR	CI	*P*	OR	CI
Regression for morbidity												
Duration	**.013**	**1.003**	**1.00–1.01**	**.006**	**1.009**	**1.00–1.02**	.916	1.000	1.00–1.00	.098	1.005	1.00–1.01
Tumor size	.351	1.090	0.91–1.31	.317	0.856	0.63–1.16	.150	1.225	0.93–1.61	.770	1.069	0.68–1.68
Simpson	.936	0.987	0.72–1.36	.331	0.737	0.40–1.37	.262	1.303	0.82–2.07	.322	1.396	0.72–2.70
Adherence	**.014**	**1.443**	**1.08–1.93**	**.021**	**1.847**	**1.10–3.12**	.566	1.180	0.67–2.08	.535	1.207	0.67–2.19
Vascularization	.496	1.165	0.75–1.81	.428	1.380	0.62–3.06	.781	1.113	0.52–2.36	.886	1.079	0.38–3.06
Regression for GTR												
Duration	.123	0.998	0.99–1.00	.417	1.005	0.99–1.02	.382	1.003	1.00–1.01	**.029**	**0.991**	**0.98–1.00**
Recurrent surgery	**.001**	**0.210**	**0.09–0.51**	.582	0.497	0.04–5.98	.090	0.278	0.06–1.22	.494	0.467	0.05–4.15
Heterogeneity	.277	0.652	0.30–1.41	.086	0.208	0.04–1.25	**.021**	**0.257**	**0.08–0.81**	.466	2.284	0.25–21.04
Adherence	**<.001**	**0.420**	**0.29–0.61**	.693	0.812	0.29–2.28	**<.001**	**0.254**	**0.13–0.51**	**.044**	**0.488**	**0.24–0.98**
Vascularization	.777	1.087	0.61–1.94	**.019**	**0.269**	**0.09–0.81**	.589	1.322	0.48–3.65	.430	1.519	0.54–4.30

GTR, gross total resection. Bold numbers highlight significant values.

### Preoperative MR Imaging

In order to assess whether preoperative standard MR imaging data allow the prediction of textural tumor parameters we calculated ratios of signal intensities of meningioma and healthy brain tissue (gray matter) in defined ROIs of 3 standard MR imaging sequences. We found only a very weak association between the T2/T1 intensity ratio of meningioma and tumor consistency (Spearman’s ρ = −0.292; *P* = .004). When comparing the T2/T1 intensity ratios between groups of meningiomas defined as distinctively hard (consistency 4 or 5) or soft tumors (consistency 1–3) the meningiomas classified as hard displayed an average tendency towards higher T2/T1 intensity ratios (1.80 vs. 1.42; *P* = .064 ANOVA) compared with those classified as softer. However, this did not reach statistical significance. No further correlations between standard MR imaging data and any textural or clinical parameters were found (see Supplementary Table).

## Discussion

Surgical resection of meningiomas continues to be the most important therapeutic option if treatment is necessary. Its success is based on the EOR and the surgical morbidity and therefore of utmost importance for the further course of disease and the patients well-being. Data on surgical variables, especially those related to the tumor texture, that may hinder complete tumor resection or affect postoperative morbidity are sparse and hardly studied in detail.

With this study we can confirm in a cohort of 300 patients with intracranial meningioma the so far implicit believe that tumor texture-related parameters have an important impact on surgical morbidity and tumor resectability. Among the texture-related parameters studied especially tumor vascularization and tumor adherence to surrounding neurovascular structures demonstrated a significant impact on outcome parameters and extend of tumor resection. An impact which was even stronger then tumor size and parameters of patients’ demographics. We found that the consistency of the meningioma itself has no statistically measurable impact on the surgical outcome parameters in the overall patient cohort. However, the consistency of a meningioma at the skull base was significantly associated with the EOR.

In the neurosurgical community, it is very well accepted that consistency (also referred to as hardness, rigidity, or stiffness) is a major limitation to achieve an optimal or complete resection of intracranial meningiomas. Therefore, many studies have focused on MR imaging techniques for the presurgical prediction of tumor consistency (reviewed in ^7,14^). However, the results of these studies are conflicting and did not provide data about the impact of meningioma consistency on surgical outcome parameters. In our study, the preoperative MR imaging using standard sequences did not provide a clinically meaningful predictive value for tumor texture. Although the tumoral T2/T1 intensity ratio reached statistical significance the association with tumor consistency was weak at best, fitting the inconsistent results in the literature.^8–10,15^ Based on the literature T2-hyperintense meningioma may tend to be of softer consistency but from a clinical viewpoint our data indicate that preoperative MR imaging using standard sequences such as T1- and T2-weighted imaging is not reliable for the prediction of tumor texture. Therefore, caution should be used when patient counseling about indication of surgery, its related morbidity and the expected EOR with regard to tumor texture is based on standard MR imaging.

While a few studies described an influence of meningioma consistency on surgical outcome these studies included either patient cohorts treated in the second half of the 20th century or mostly skull base meningioma with small patient numbers.^5,16–19^ Furthermore, in these studies the assessment of tumor consistency was not standardized and purely descriptive (eg, hard vs. soft) with considerable variations. In our study, we used an adaption of a standardized 5-point grading system introduced by Zada et al.^12^ that was based on the instruments used to resect the tumor. Surprisingly, we found that the consistency of meningiomas had no influence neither on surgical morbidity nor on EOR in the overall patient cohort. This is in contrast with a previous study reporting a higher EOR (lower Simpson grade) in meningiomas with a soft to intermediate consistency using a 3-point grading system. It is reasonable to speculate that the “hardness” of a meningioma is not that relevant in certain anatomical locations such as the convexity, where the surgical resection technique can easily adapted to the consistency and a kind of hardness may even foster the resection. However, analyzing meningiomas at the skull base region with its more critical and densely localized neurovascular structures revealed a significant impact of tumor consistency on the EOR which is in line with a previous report on a series of petroclival meningiomas.^6^ A meningioma with a “hard” consistency at the skull base was less likely resected completely and resulted in a higher Simpson grade. Tumor remnants are usually only left behind in order to avoid surgical morbidity and therefore we found no association of meningioma consistency with surgical deficits in our study.

Tumor texture includes not only the consistency but also other important parameters like tumor vascularization and adherence to the surrounding brain parenchyma and neurovascular structures. In our study, we found that the tumor vascularization had a significant impact on surgical morbidity and EOR in the overall patient cohort. Notwithstanding, tumor adherence displayed the most significant influence of all assessed textural features on surgical morbidity and extend of tumor resection, being an independent risk predictor in the overall patient cohort. While we found no significant association with surgical morbidity in the skull base group as opposed to the findings by Little et al.,^6^ the EOR was significantly reduced in meningiomas displaying a high adherence similar to previous results.^6^ These differences may be the result and confirm to some extend that during surgery the neurosurgeon naturally adapts the resection technique according to the intraoperative findings such as consistency including hardness and adherence. This may be much more in cases of skull base meningiomas were important neurovascular structures are often in near vicinity, displaced or even encased by the tumor than in other anatomical locations therefore avoiding the risk of postoperative morbidity by reducing the EOR. This underlines that relevant consistency parameters for surgical outcome need to be established in order to develop appropriate imaging techniques for the presurgical planning and risk prediction especially in neuroanatomical challenging locations such as the skull base.

### Limitations

One of the major obstacles of studying texture-related tumor parameters is the lack of quantitative methods for their objective assessment in the intraoperative setting. In order to move further beyond the so far used simple classifications such as “hard” versus “soft” we used a standardized questionnaire using a grading system based on intraoperative aspects and tool necessary for the tumor resection which depend on the tumor texture. Nevertheless, the subjective impression of the surgeon is still a major factor. Despite the large cohort of 300 patients with meningioma it has to be pointed out, that statistical power is reduced in the subgroup calculations. However, we checked thoroughly whether the subgroup results showed equidirectional tendencies.

## Conclusions

Our analysis demonstrates that tumor texture has a significant impact on the surgical management of meningioma. Here, we provide data that tumor vascularization and adherence are highly significant factors influencing surgical outcome whereas the influence of tumor consistency has less impact than previously thought. Preoperative prediction of tumor texture is therefore required for optimizing the surgical strategy and risk assessment. Current standard MR imaging is not sufficient for a reliable prediction of tumor consistency and refined MR imaging and its analysis needs to be developed.

## Supplementary Material

vdaa113_suppl_Supplementary_Material
