# Supplementary material for: Features of tumor texture influence surgery and outcome in intracranial meningioma
Source: Neurooncol Adv. 2020 Sep 10;2(1):vdaa113. doi: 10.1093/noajnl/vdaa113 (PMC7586142; doi:10.1093/noajnl/vdaa113)
Supplement: vdaa113_suppl_Supplementary_Material [file vdaa113_suppl_supplementary_material.docx]

| Correlation with MRI Findings | | | | | | | |
| --- | --- | --- | --- | --- | --- | --- | --- |
| Spearman's correlation | | | **Change in mRS** | **WHO grade** | **Tumor consistency** | **Tumor vascularization** | **Tumor adherence** |
|  | T1-C+ intensity M/GM | **ρ** | -0.177 | 0.042 | -0.057 | 0.151 | -0.004 |
|  |  | **p-val.** | 0.054 | 0.650 | 0.535 | 0.101 | 0.963 |
|  | T1 intensity M/GM | **ρ** | -0.163 | -0.069 | -0.121 | 0.040 | -0.110 |
|  |  | **p-val.** | 0.089 | 0.471 | 0.208 | 0.676 | 0.252 |
|  | T2 intensity M/GM | **ρ** | -0.031 | 0.043 | -0.189 | 0.069 | 0.168 |
|  |  | **p-val.** | 0.752 | 0.658 | 0.051 | 0.478 | 0.083 |
|  | T2/T1 intensity M | **ρ** | -0.037 | 0.086 | **-0.292^**^** | 0.123 | -0.151 |
|  |  | **p-val.** | 0.719 | 0.399 | **0.004** | 0.227 | 0.139 |

T1 = T1-wheigted MRI sequence; T1C+ = Contrast enhanced T1-wheigted MRI sequence ; T2 = T2-wheighted MRI sequence; M = meningioma; GM = grey matter; ρ = Spearman’s rank correlation coefficient; ** correlation is significant at the 0.01 level (2-tailed); p-val. = p-value
